# In the Eye of the Storm: Entrepreneurs and Well-Being During the COVID-19 Crisis

**DOI:** 10.1177/10422587211057028

**Published:** 2021-12-14

**Authors:** Mikaela Backman, Johannes Hagen, Orsa Kekezi, Lucia Naldi, Tina Wallin

**Affiliations:** 1 Center of Entrepreneurship and Spatial Economics (CEnSE), 4161 Jönköping International Business School, Jönköping University, Sweden; 2 Swedish Institute for Social Research (SOFI), Stockholm University, Stockholm, Sweden

**Keywords:** well-being, entrepreneurs, crisis, subjective age, location, **JEL-codes:** C83, I31, J10, L26.

## Abstract

In this paper, we examine the impact of the COVID-19 pandemic on the well-being of entrepreneurs. We surveyed a representative sample of Swedish entrepreneurs and wage employees at different stages of the COVID-19 pandemic. The survey data, combined with register data, show that the COVID-19 outbreak has a negative effect on the well-being of entrepreneurs in terms of increased perceived stress. However, this negative effect is weaker for entrepreneurs who feel younger than their chronological age and entrepreneurs who are geographically distant from the epicenter of the crisis.

## Introduction

Entrepreneurs work long hours, juggle different roles, and operate under uncertainty and with limited resources (Aldrich & Martinez, 2007; Wagner, 2003). They also pay an emotional (Cacciotti et al., 2016; De Cock et al., 2020) and financial price if the venture fails or underperforms (Gimeno et al., 1997). External uncertainty and economic shocks, such as the COVID-19 outbreak, may thus be particularly stressful for entrepreneurs since such events can amplify existing stressors, in particular financial uncertainties, that are known to be detrimental to entrepreneurs’ well-being (Schonfeld & Mazzola, 2015; Kollmann et al., 2019; Stephan, 2018). For instance, Patel and Rietveld (2020) found that during the COVID-19 pandemic, entrepreneurs have experienced greater psychological distress than employees due to financial insecurity. Other recent studies have shown that lack of financial resources during COVID-19 has been particularly problematic for entrepreneurs because the crisis has dramatically reduced the availability of early-stage seed investments (Brown & Rocha, 2020), and because start-ups, to a large extent, have been excluded from policy measures and support programs (Kuckertz et al., 2020).

This begs the question; how can entrepreneurs maintain their well-being in the face of crises and shocks? Research has identified several approaches (Uy et al., 2013) that entrepreneurs may employ to reduce stress and improve their health and well-being. For example, Murnieks et al. (2020) demonstrated that mindfulness practice and sleep can reduce exhaustion in an entrepreneurial context, and Wach et al. (2020) found that the ability to recover from stress during non-working hours may be impaired if one does not succeed in detaching from work. In addition, Kollmann et al. (2019) introduced prior entrepreneurial experience as a decisive factor in determining an entrepreneur’s recovery and well-being, while Cardon and Patel (2015) showed that having positive affectivity may limit the detrimental effects of stress.

Many of these findings build on the notion that psychological detachment *from work* may allow entrepreneurs to recover, thereby reducing strain and rebuilding well-being (Kollmann et al., 2019). In this paper, we extend this line of research by suggesting two novel forms of psychological detachment *from the crisis*. These types of psychological detachment—the *illusion of age* (feeling younger than one’s chronological age) and the *illusion of space* (being located far from the epicenter of the pandemic)—have the potential to protect entrepreneurs from the negative effects of the COVID-19 outbreak. These illusions relate to two critical aspects of the pandemic: the fact that the mortality rate increases with age and the risk of infection decreases with geographical distance from the virus’ epicenter. We theoretically propose and empirically assess: (i) how the COVID-19 pandemic has negatively affected entrepreneurs’ well-being, (ii) how feeling younger than one’s chronological age, and (iii) how the distance from the epicenter of the crisis might mitigate the negative effects of the COVID-19 pandemic on entrepreneurs’ well-being.

We test our predictions using a unique dataset, which combines register data with survey data on a representative sample of new entrepreneurs before and after the initial phase of the COVID-19 outbreak in Sweden during the spring of 2020. Our empirical strategy exploits the fact that the respondents answered the survey at different stages of the COVID-19 pandemic, starting in February—which was 1 month before the government-imposed restrictions—and continuing until May 2020. In addition, in Sweden, which is the country of investigation of this study, at the outbreak of COVID-19 the government imposed nationwide and not region-specific regulations and restrictions. In short, we find that entrepreneurs’ well-being, as measured by perceived stress, worsened during the COVID-19 crisis, but this effect is alleviated if the entrepreneur reported feeling younger than their chronological age and is not located in the epicenter of the COVID-19 pandemic.

Given the limited knowledge on entrepreneurial well-being during major and sudden crises, our study advances research by offering empirical evidence of the negative effect of the COVID-19 outbreak on stress perceived by entrepreneurs. In addition, we identify entrepreneur-specific factors—related to critical aspects of the crisis—that provide a buffering effect for the perceived stress. By theorizing about psychological detachment from the crisis as a mechanism that can help entrepreneurs to alleviate stress, we make two contributions to the entrepreneurship literature. First, we shed new light on how some entrepreneurs can maintain positive functioning despite adversity, hence adding to the research on how entrepreneurs build resilience and react after major crises (Farny et al., 2019; Shepherd and Williams, 2014, 2020). Second, our focus on detachment from the crisis supplements existing work that has largely focused on the mechanisms for detaching from work (Kollmann et al., 2019).

## Theory and Hypotheses

Following the literature on subjective well-being, especially hedonic well-being, we conceptualize well-being as: (i) an evaluative component—life satisfaction—and (ii) the absence of a negative component—in terms of perceived stress (Ryan & Deci, 2001; Stephan, 2018). Entrepreneurship is understood in terms of the creation of new ventures (Gartner, 1985); thus, entrepreneurs are defined as founders of new ventures.

Our theoretical framework builds on the stressor-detachment model (Sonnentag & Fritz, 2015), which emphasizes the role of psychological detachment in stressor-strain relationships, and social psychology literature on positive illusions (Taylor, 1983; Taylor & Brown, 1988).

Stressors consist of stressful work-related factors that can lead to poor psychological well-being. While the model by Sonnentag and Fritz (2015) focuses on stressors experienced by employees in the work environment—such as work-overload or work complexity—we argue that the outbreak of COVID-19 can function as a work-related stressor for entrepreneurs. The pandemic and ensuing government restrictions created new constraints on entrepreneurs’ business activities, preventing them from doing their jobs successfully (Kollmann et al., 2019).

Psychological detachment refers to the extent to which individuals keep a mental or psychological distance from work stressors. As such, psychological detachment can attenuate the negative effects of stressors on well-being (Sonnentag & Fritz, 2015). Lack of psychological detachment is characterized by continuous mental engagement with stressors, such as the inability to stop thinking about work stressors even during non-work time (Wach et al., 2020).

While entrepreneurs might psychologically distance themselves from the adversity of the COVID-19 outbreak in different ways, studies in social psychology have shown that positive illusions—and not accurate perceptions—might promote such distancing (Taylor, 1996). Thus, positive illusions may help entrepreneurs psychologically detach from the COVID-19 outbreak and the corresponding work-related stressors. We first develop our baseline hypothesis regarding the negative effect of COVID-19 on the well-being of entrepreneurs and then argue that two positive illusions—*illusion of age* and *illusion of space*—reduce this negative effect.

### Well-Being and Entrepreneurs in Times of Crises

As a major and sudden crisis, the COVID-19 outbreak can increase the number of financial uncertainties that entrepreneurs might encounter, intensifying the stressors that are known to be detrimental to their well-being (Schonfeld & Mazzola, 2015). Entrepreneurs are emotionally and financially involved in their ventures (Shepherd et al., 2009; Jenkins et al., 2014) and at times even personally liable for the firm’s debts. They also have limited access to safety-net programs compared to employees (Wolfe & Patel, 2021). In a crisis, entrepreneurial stressors are accentuated by a simultaneous plummeting of demand for goods and services and increasingly difficult access to financial resources. With specific reference to the COVID-19 outbreak, Patel and Rivtveld’s (2020) study shows that economic uncertainty induced by the pandemic hit entrepreneurs more harshly than employees, thus increasing their psychological distress. In addition, the COVID-19 outbreak has negatively impacted the availability of financial resources as financiers become more reluctant to provide capital to new ventures (Brown & Rocha, 2020; Brown et al., 2020; Demirgüç-Kunt et al., 2020).

Besides the potential loss of income and risk of failure, financial uncertainties following the COVID-19 outbreak might have been perceived as stressful due to the responsibility that entrepreneurs have for employees and other stakeholders. This is well exemplified by one of the entrepreneurs from Doern’s (2021: 4) diary study who reported that “[n]egatively dealing with people, staff and customers alike has been unbelievably, unbearably stressful.”

In addition, uncertainty about whether these shifts are permanent or temporary also limits entrepreneurs’ ability to deal with these stressors (Donthu & Gustafsson, 2020; Sheth, 2020). Taken together, these arguments suggest that COVID-19 has a negative impact on the well-being of entrepreneurs.

## Baseline Hypothesis: The COVID-19 Pandemic has a Negative Effect on the Well-Being of Entrepreneurs

### Illusion of Age

The risk of severe illness and the mortality rate from COVID-19 both increase with chronological age, creating a clear demographic segmentation in the course of the disease. We hypothesize that entrepreneurs who feel younger than their chronological age are more able to detach from the outbreak and the corresponding stressors.

Subjective age is connected to a person’s self-understanding and self-knowledge, and in “adulthood, this self-representation becomes an integral part of a person’s overall self-concept and identity” (Shrira et al., 2018: 2). Some scholars have also connected subjective age to the attempts made by individuals to distance themselves from a negatively stereotyped group (Weiss & Lang, 2012).

Although subjective age might be a marker of one’s physical and physiological functioning, research shows that adopting various cognitive strategies, such as making selective social comparisons, allows an individual to see their own situation in a more positive light (Heckhausen & Krueger, 1993; Uotinen et al., 2006). Feeling younger provides, for example, a positive social identity (Weiss & Lang, 2012) and perceived control in domain-specific areas (Levy, Slade, & Kasl, 2002). As such, feeling younger can predict positive outcomes.

Additionally, empirical studies have documented that feeling younger than one’s chronological age is associated with greater life satisfaction (Wurm et al., 2008), longer lives (Kleinspehn-Ammerlahn et al., 2008; Levy, Slade, Kunkel, & et al., 2002), better overall health (Levy, Slade, & Kasl, 2002), and improved physical abilities (Stephan et al., 2013). In the workplace context, younger subjective age is associated with increases in productivity (Kunze et al., 2015; Rodríguez-Cifuentes et al., 2018), adaptability to new work situations (Rudolph & Baltes, 2017), perceived control and motivation (Shane et al., 2019), job crafting (Nagy et al., 2019), and the probability of starting new ventures (Kautonen et al., 2014, 2015). In addition, Gielnik et al. (2018) found that a longer perceived remaining work-life is positively related to entrepreneurial intentions.

Besides these direct beneficial effects, research has also shown that subjective age can be important in alleviating the effects of stressful events and traumas, such as cancer (Boehmer, 2007) and Post-Traumatic Stress Disorder (Palgi, 2016; Shrira et al., 2018). Additionally, these studies argued that individuals who perceive themselves as younger than their chronological age find it easier to dissociate themselves from the trauma burden.

In the specific case of entrepreneurs in the aftermath of the COVID-19 outbreak, feeling younger than their chronological age might help entrepreneurs with the process of unwinding from the crisis and the intensified work-related stressors. Thus, we argue that an “illusion of age” in the sense of feeling younger than one’s chronological age can help entrepreneurs psychologically detach from COVID-19 and the corresponding work-related stressors, thereby alleviating the negative effects of the outbreak on their well-being.

Hypothesis 1: The negative relationship between the COVID-19 pandemic and the well-being of entrepreneurs is weaker for entrepreneurs who feel younger than their chronological age.

### Illusion of Space

The outbreak of COVID-19—like many other major and unexpected crises (Rehdanz et al., 2015)—has an important spatial component. In most countries, COVID-19 spread from one or several local epicenters with subsequently varying infection and mortality rates across geographical locations.^1^ In contrast, the geographical component of the economic effects of the initial phase of the pandemic was much weaker, partly because the regulations and restrictions imposed at the time were nationwide rather than regional. Thus, increased physical distance between an entrepreneur and the local epicenter of the COVID-19 outbreak could create an “illusion of space” that mitigates the perception of the severity of the crisis and weakens the effect of the crisis on the entrepreneur’s well-being.

The role of spatial dependencies and physical distance is emphasized by Tobler (1970), who stated that the physical distance between two factors dictates the strength of the relationship. Proximity induces a stronger relationship between two factors, ceteris paribus (Dorigo & Tobler, 1983; Joo et al., 2017). Tobler’s arguments mainly build on the spatial distribution of economic phenomena, disregarding the psychological aspects of individuals and how they react to a crisis. Research in psychology predicts that individuals habitually use psychological distance to assess the likelihood of the occurrence of an event and how the event relates to their own circumstances, for example, becoming sick and/or suffering financially from the COVID-19 outbreak. Psychological distance can be categorized into four often interrelated distances: temporal (whether the event happens in the near or distant future), hypothetical (whether the event is likely to be true or close to the individual’s reality), social (whether the event is socially distant, i.e., happening to friends or family), and physical (whether the event is geographically close by) (Trope & Liberman, 2010; Bar-Anan et al., 2007). Abridged physical distance, which is the only quantifiable type of psychological distance, has been observed to increase an individual’s assessment that an event, either positive or negative, is likely to occur (Wyer et al., 2014). Physical distance also has an amplified effect on the other types of psychological distance (Liberman et al., 2007; Wyer et al., 2014). Thus, enlarged physical distance enables an individual to psychologically detach from a stressful event.

Consequently, we hypothesize that being geographically distant from the local epicenter of the COVID-19 outbreak enables entrepreneurs to mentally distance themselves from the crisis and better manage work-related stressors, thereby attenuating the negative effects of such stressors on their well-being.

Hypothesis 2: The negative relationship between the COVID-19 pandemic and the well-being of entrepreneurs is weaker for entrepreneurs who are geographically distant from the local epicenter of the outbreak.

## Empirical Analysis

In the remainder of the paper, we first provide a brief description of the COVID-19 outbreak in Sweden. We then describe the survey that we used to test our hypotheses, including sample restrictions and key variables. After displaying our empirical models, we present and discuss the main results followed by several robustness tests and post-hoc analyses.

### The COVID-19 Outbreak in Sweden

The Swedish government’s strategy for handling the spread and impact of COVID-19 in its initial phase differed from the methods employed by many other countries. The stated objective of the Swedish government was to “flatten the curve” by influencing the pace of the spread to achieve a more evenly distributed number of infected and severely ill people over time. The Swedish government used several different measures to attempt to slow the spread of COVID-19 but did not, unlike other countries, resort to lockdowns.^2^ The economic decline in the initial phase of the COVID-19 outbreak reveals similar patterns in aggregate consumer spending in Sweden compared to countries enforcing stronger restrictions, such as lockdowns (Sheridan et al., 2020). Businesses in Sweden thereby provide a unique context to study the effects of a major crisis on entrepreneurs’ well-being as they continued to operate during the pandemic while still experiencing similar economic consequences as businesses in other countries.

The first death from COVID-19 in Sweden was reported on March 11, 2020, in Stockholm. Consequently, several nationwide recommendations and restrictions were imposed between March 12 and March 19.^3^ The imposition of restrictions combined with the first death and a subsequent increase in the number of COVID-19 cases represent a clear shift in the way the disease was perceived by the public authorities and society. The main peak of the outbreak appeared in the first half of April 2020. In our empirical analysis, we define week 12 (starting on March 16) as our first “post-COVID-19” observation.

As of September 2021, just above 14,700 individuals have died from COVID-19 in Sweden, about 3800 of whom died during our survey period (week 8–week 20 of 2020). The demographic profile of the deceased follows the same pattern seen in other countries—men and older individuals are over-represented.^4^ Stockholm County, the epicenter of the outbreak in the spring of 2020, recorded the highest number of deaths per capita. In May 2021, the county accounted for approximately 30% of all deaths in Sweden, and in the initial phase of the pandemic, it accounted for about 40% of all deaths. These statistics show a clear overrepresentation, as Stockholm County accounts for less than 25% of the population.^5^

In Sweden, the regulations and restrictions initially imposed by the government during this period were nationwide and not specific to a region such as the epicenter. Therefore, the financial stress caused by disruptions to economic activity was likely experienced at similar levels across all of Sweden, which can be empirically observed in the data. Although Stockholm was at the medical epicenter of the virus, the economic impacts experienced in Stockholm have not been worse than in other regions of Sweden. For example, the corporate bankruptcy rate in 2020 in Stockholm was no higher than the national average (UC, 2020). In terms of the share of workers receiving government-financed short-time work allowance, Stockholm’s share was just above the national average of 18.1% (Ekonomifakta, 2020).

### The Survey

Our empirical analysis is based on two jointly designed surveys that were sent out by Statistics Sweden between February and May 2020 (week 8–week 20). One survey was sent to a sample of entrepreneurs, and the other was sent to a matched sample of non-entrepreneurs. The sampling for the entrepreneur survey was done in two steps. First, we sampled the universe of ventures that were started in Sweden in 2016 (4 years prior to the survey). Second, we identified the (main) founder of these new ventures. Since prior research shows that the effects of stressors on entrepreneurs’ well-being can be moderated by previous entrepreneurial experience (Kollmann et al., 2019), we focus only on novice entrepreneurs to limit the confounding effect of prior entrepreneurial experience.

The sample of entrepreneurs was stratified based on two age groups: 40–49 and 50–79. The cut-off point of 40 years of age was made based on prior research on subjective age (Westerhof & Barrett, 2005). This literature suggests that the inclination to feel younger than one’s chronological age is likely to commence later in the life cycle (Ye & Post, 2020; Rubin & Berntsen, 2006). In addition, individuals in their adulthood have more stable subjective age identities (Barrett & Montepare, 2015).

The survey was sent out to a random sample of 1500 entrepreneurs in the 40–49 age group and 3000 entrepreneurs in the 50–79 age group. For the non-entrepreneur survey, we sampled 2500 individuals from ages 40–79 who had never been registered as business owners in Statistics Sweden’s data. The response rates for entrepreneurs and non-entrepreneurs were 29.6% (1332 individuals) and 40.1% (1003 individuals), respectively. For the non-entrepreneurs, we restricted the sample to those who were employed in 2016, henceforth referred to as the employee sample. 4647 individuals who received either of the two surveys did not reply, the majority (97%) for unknown reasons. Respondents could answer the surveys by either mailing in a paper version or answering it online. A letter was sent out between February 17th and 21st (week 8) with information on how to complete the survey online, as well as instructions for mailing in the paper version. The survey closed on May 17th (week 20).

The survey responses were matched to register data from Statistics Sweden. Access to register-based data has two main advantages: first, we could reduce the number of background questions in the survey and instead use detailed, individual-level information available in the register data, and second, we could examine the extent of—and also address—sample selection bias (see the next section).

**Figure A1:**
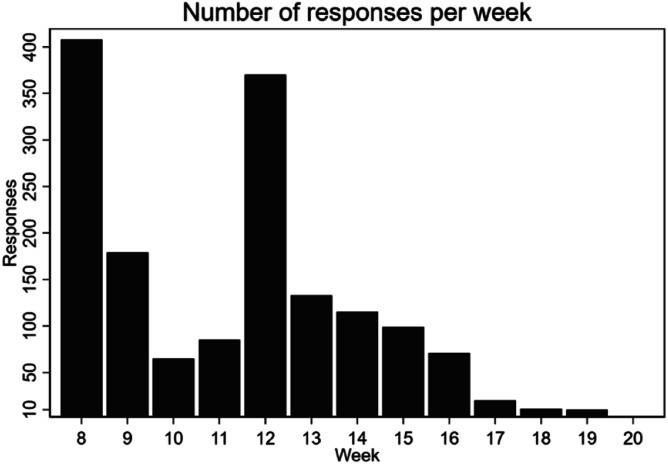
Number of survey responses per week.

Our empirical strategy exploits the fact that respondents completed the survey at different stages of the COVID-19 pandemic. The number of survey responses per week is provided in the appendix (Figure A1). Approximately 47% of the respondents in the entrepreneur sample, completed the survey before the virus outbreak (i.e., no later than week 12).

### Sample Selection

For our results to be representative of the targeted population, that is, founders of new ventures in Sweden and employees, we need to account for the differences between the respondents and the targeted population. We do so by using the entropy balancing technique suggested by Hainmueller (2012). With this method, weights are constructed such that the weighted sample has the means of the observed covariates that exactly correspond to the means in the target population. We use the register-based covariates presented in the Appendix (Table A1) for the entropy balancing. These covariates are included in the set of control variables in the empirical estimations.

### Variables

*Dependent variables:* We focus on two dimensions of well-being: stress and life satisfaction. Each well-being variable is constructed as an average of the response to several interrelated survey questions. Following prior research (e.g., Chiu et al., 2005), we measure perceived stress using a four-item version of the Perceived Stress Scale initially proposed by Cohen et al. (1983) and Cohen and Williamson (1988) and often used in the empirical literature (Baron et al., 2016; Kibler et al., 2019; Lerman et al., 2020; Chiu et al., 2005). Participants responded to the following items: (1) Feeling nervous and stressed; (2) Feeling that you do not have time to finish what you have to do; (3) Being angry about things that have happened that are beyond your control; and (4) Feeling that you have had many problems that you have not been able to take care of. Participants rated the items on a five-point Likert scale anchored by 1 = “Strongly disagree” and 5 = “Strongly agree.” We take the average of the responses to the four questions as the level of perceived stress. Cronbach’s alpha for this scale is 0.845.

To measure life satisfaction, we use the Satisfaction with Life Scale (SWLS), which was initially proposed by Diener et al. (1985) and captures the following items: (1) In most ways my life is close to my ideal; (2) The conditions of my life are excellent; (3) I am satisfied with my life; (4) So far, I have gotten the important things I want in life; and (5) If I could live my life over, I would not change anything. This scale has been extensively used in the literature to measure life satisfaction in different countries, including Sweden (Pavot and Diener, 1993, 2008; Glaesmer et al., 2011; Hultell & Gustavsson, 2008). Participants rated the items on a five-point Likert scale anchored by 1 = “Strongly disagree” and 5 = “Strongly agree.” Just as with the measure of perceived stress, we take the average of the responses to the five questions. Cronbach’s alpha of this scale is 0.883.

*Main independent variables of interest:* Our main independent variables of interest are: COVID-19, Stockholm, and Feel younger. The first two are straightforward. COVID-19 takes the value of 1 if the individuals answered the survey on or after week 12, and Stockholm takes the value of 1 if the respondents live in Stockholm County.

Regarding the third variable, we asked the respondents in the survey about their subjective age, that is, how old they feel they are. We create a binary variable, *Feel younger*, that takes the value of 1 if the individual feels strictly younger than his or her chronological age and 0 otherwise. We use this as our central measure of subjective age. About 96% report a different subjective age than their chronological age, with about 77% feeling younger. The corresponding share among the employees is 78% (see Table 1). Several studies use various categorizations rather than continuous measures of subjective age (c.f. Boehmer (2007); Infurna et al. (2010); Nagy et al. (2019); Rodríguez-Cifuentes et al. (2018)). Using a binary measure of subjective age has several advantages. First, it acknowledges the possibility that feeling younger or older may not be a symmetric or linear cognitive process (Kwak et al., 2018; Kotter-Grühn & Hess, 2012; Weiss & Lang, 2012). In other words, the association between a given increase in subjective age, on the one hand, and stress or life satisfaction, on the other hand, could vary across the subjective age distribution. Second, a binary classification reduces the influence of unreasonably large differences between subjective and chronological age (“outliers”). The third advantage is related to a general concern about the stability of subjective age. While previous studies find that subjective age is a rather stable attribute over time (Cleveland et al., 1997; Uotinen et al., 2006; Kleinspehn-Ammerlahn et al., 2008; Kotter-Grühn et al., 2015), some fluctuations can occur within a short period (Kotter-Grühn et al., 2015; Goecke & Kunze, 2020).^6^

**Table 1.** List of Variables Included in the Empirical Estimations.

Variables	Measured as	Mean	Sd	Mean	SD
		Entrepreneurs		Employees	
Dependent variables					
Stress*	The average of responses to four questions; answer choices are ranked 1–5. See the section Variables in the text for details	2.308	0.884	2.415	.952
Life satisfaction*	The average of responses to five questions; answer choices are ranked 1–5. See the section Variables in the text for details	3.948	0.781	3.839	.833
Independent variables					
COVID-19	Dummy = 1 if the individual answered the survey after week 12	0.551	0.498	0.504	0.5
Feel younger*	Dummy = 1 if the subjective age is lower than chronological age	0.765	0.424	0.78	0.415
Subjective age*	((Chronological age – subjective age)/chronological age)	0.102	0.151	0.111	0.178
Stockholm**	Dummy = 1 if the individual lives in Stockholm County	0.313	0.464	0.222	0.416
Age**	Age of the individual when creating the venture	55.181	8.19	53.824	8.883
Female**	Dummy = 1 if the individual is female	0.362	0.481	.561	0.497
Education**	Level of education (six categories pre-defined by Statistics Sweden) 1) Less than 9 years of schooling 2) 9 years of schooling 3) Secondary education 4) Post-secondary education shorter than two years 5) Post-secondary education longer than two years 6) PhD education	4.257	1.055	3.856	1.138
Incorporated*	Dummy = 1 if the firm is an incorporated business	0.482	0.5		
Married**	Dummy = 1 if the individual is married or in a registered partnership	0.636	0.481	0.558	0.497
Income**	Earned yearly income in 2016, 100s of SEK (in logs)	4914	4802.4	3842.6	2272.9
Sick**	Dummy = 1 if the individual has received sickness benefits anytime during 2012–2016	0.273	0.446	0.306	0.461
Industry Experience*	Previous industry experience. Three categories: no/limited, moderate, extensive	2.252	0.834		
Web*	Dummy = 1 if the survey was answered on the web	0.644	0.479	0.645	0.479
Density**	Population density in the municipality the individuals reside (population/km^2^)	1001.2	1707.8	732	1429.8

*Survey data. ** Register data from Statistics Sweden.

To explore the stability of subjective age as a measure in more detail, we conducted an independent online survey with a longitudinal design on a representative sample of Swedish citizens aged 40–80. The details of the additional survey, along with further tests of the stability of subjective age, are presented and discussed in the post-hoc analyses section. In a robustness analysis, we also use a discrepancy measure of subjective age calculated as the percentage gap between chronological age and subjective age (Rubin & Berntsen, 2006; Ye & Post, 2020). As opposed to an absolute subjective age gap, a percentage gap is comparable across different ages (Rubin & Berntsen, 2006).^7^

*Control variables:* We control for several characteristics that may impact individuals’ well-being as the pandemic unfolded. The individual-level variables are taken from the register data, which have been merged with the survey responses, and include variables that previous studies have documented as related to well-being: chronological age (Diener et al., 1999; Nikolaev et al., 2020), gender (Stevenson & Wolfers, 2009; Kibler et al., 2019), educational attainment (Ross & Van Willigen, 1997; Nikolaev & Rusakov, 2016; Bhuiyan & Ivlevs, 2019), marital status (Nikolaev et al., 2020), health proxied through sick leave absence (Graham, 2008; Shields & Price, 2005), income (Kahneman & Deaton, 2010; Clark et al., 2008), and industry (1-digit NACE). The choice of response method may be correlated with the timing of the response and individual characteristics that are not captured by the variables described above. Thus, we also control for whether the individual submitted the survey responses online or through mail. Two additional self-reported control variables are added in the estimations for the entrepreneur sample. These variables capture previous work experience in the industry to which their current firm belongs and the organizational form of the firm.

At the regional level, we control for population density in the municipality where the respondents live. There are two reasons for this. First, we want to account for the possibility that living in more densely populated areas may have direct implications on individual well-being and stress. Second, we want to rule out the possibility that the potential effect of living in Stockholm on well-being is driven by a general “big city” effect rather than the hypothesized effect of living in the vicinity of the local epicenter.

Since our dependent variables and the variable *Feel younger* originate from the same survey, our results might be inflated by common source variance (CMV). Following Lindell and Whitney (2001), we evaluated the presence of this bias using a market variable approach. We first identified a marker variable, which should be theoretically not related and correlated to the other variables originating from the same survey. We selected as marker variable whether the respondents reported having *Sibling entrepreneurs* or not. We found that this marker variable was not statistically correlated to the survey-based variables in our model. Thereafter, we conducted a partial correlation analysis by subtracting the correlation between the market variable and the focal variables from the correlations among the focal variables. We found the proposed correlations still retain significance after the adjustment for CMV contamination. To further assess if we face a common source bias we follow the suggestion by Podsakoff et al. (2003) and use the identified marker variable as a control variable in our estimations. The inclusion of this variable did not change our results in any substantial way. Taken together, these results indicate that common method bias is not a serious concern in our study.

Table 1 lists and presents descriptive statistics for all the variables that are included in the empirical estimations for the sample of entrepreneurs, as well as our control group of employees.

Table 2 provides the correlation matrix of the variables used in the empirical estimations. Two bivariate correlations which could be considered borderline cases are the dummy variable for Stockholm and population density as well as web and the COVID-19 dummy. The first is not surprising as Stockholm County is the most densely populated region in Sweden. Regarding the second, to ensure that it does not affect our results, we have run the model with and without the dummy for web, and the results are consistent. Even so, we calculated the variance inflation factor (VIF) for all independent variables of our model (3a and 3b) in Table 3 to check for potential multicollinearity. None of the variables in our model have a VIF greater than 10, suggesting that multicollinearity is not an issue with our data.

**Table 2.** Correlation Matrix.

	(1)	(2)	(3)	(4)	(5)	(6)	(7)	(8)	(9)	(10)	(11)	(12)	(13)	(14)	(15)
(1) Stress	1.000														
(2) Life Sat.	−0.421	1.000													
(3) COVID-19	0.104	−0.034	1.000												
(4) Feel younger	−0.126	0.101	0.005	1.000											
(5) Stockholm	−0.006	−0.001	0.051	0.005	1.000										
(6) Age	−0.353	0.185	0.009	0.118	−0.070	1.000									
(7) Female	0.142	0.006	0.032	−0.005	0.010	−0.106	1.000								
(8) Education	0.019	0.112	−0.028	−0.013	0.098	−0.069	0.120	1.000							
(9) Incorporated	−0.026	0.078	−0.055	0.002	0.109	−0.054	−0.153	0.119	1.000						
(10) Married	−0.061	0.175	0.024	−0.011	0.006	0.098	−0.084	0.075	0.030	1.000					
(11) Income	0.004	0.060	−0.068	−0.003	0.062	−0.247	−0.056	0.125	0.274	−0.017	1.000				
(12) Sick	0.121	−0.130	0.059	−0.050	−0.037	−0.108	0.180	−0.042	−0.136	−0.058	−0.032	1.000			
(13) Industry Exp.	−0.109	0.101	−0.035	0.001	0.052	0.185	−0.145	0.080	0.164	0.053	0.146	−0.120	1.000		
(14) Web	−0.061	−0.000	−0.662	0.022	0.059	−0.108	−0.072	0.050	0.043	−0.019	0.092	−0.044	0.003	1.000	
(15) Density	0.016	0.025	−0.004	−0.010	0.636	−0.075	−0.004	0.207	0.154	−0.007	0.087	−0.056	0.076	0.081	1.000

All correlations above 0.1 are statistically significant at 0.05%.

**Table 3.** Main Results for the Sample of Entrepreneurs.

	Stress	Stress	Stress	Stress	Life Satisfaction	Life Satisfaction	Life Satisfaction	Life Satisfaction
	(Base)	(1a)	(2a)	(3a)	(Base)	(1b)	(2b)	(3b)
COVID-19		0.255***	0.463***	0.158*		−0.076	−0.137	−0.011
(C)		(0.075)	(0.126)	(0.086)		(0.069)	(0.116)	(0.079)
Feel younger		−0.183***	−0.035	−0.183***		0.054	0.011	0.054
(FY)		(0.067)	(0.098)	(0.067)		(0.062)	(0.090)	(0.062)
Stockholm		−0.058	−0.056	−0.217**		−0.061	−0.062	0.046
(S)		(0.085)	(0.085)	(0.110)		(0.078)	(0.078)	(0.101)
C*FY			−0.274**				0.080	
			(0.133)				(0.122)	
C*S				0.283**				−0.190*
				(0.124)				(0.114)
Age	−0.036***	−0.034***	−0.034***	−0.034***	0.019***	0.019***	0.019***	0.019***
	(0.004)	(0.004)	(0.004)	(0.004)	(0.003)	(0.003)	(0.003)	(0.003)
Female	0.110*	0.125*	0.123*	0.118*	0.091	0.089	0.089	0.093
	(0.064)	(0.066)	(0.066)	(0.066)	(0.058)	(0.061)	(0.061)	(0.061)
Education	0.018	−0.001	0.003	−0.001	0.014	0.010	0.009	0.011
	(0.030)	(0.030)	(0.030)	(0.030)	(0.027)	(0.028)	(0.028)	(0.028)
Incorporated	0.052	0.050	0.048	0.046	0.060	0.053	0.054	0.055
	(0.061)	(0.063)	(0.063)	(0.063)	(0.056)	(0.058)	(0.058)	(0.058)
Married	−0.126**	−0.106*	−0.104*	−0.110*	0.242***	0.226***	0.226***	0.229***
	(0.058)	(0.060)	(0.059)	(0.059)	(0.053)	(0.055)	(0.055)	(0.055)
Income	−0.048***	−0.043**	−0.043**	−0.042**	0.056***	0.055***	0.055***	0.054***
	(0.018)	(0.018)	(0.018)	(0.018)	(0.017)	(0.017)	(0.017)	(0.017)
Sick	0.015	0.148**	0.139**	0.153**	0.006	−0.174***	−0.171***	−0.177***
	(0.035)	(0.066)	(0.066)	(0.066)	(0.032)	(0.061)	(0.061)	(0.060)
Industry Exp.	0.163**	0.007	0.008	0.004	−0.190***	0.015	0.015	0.017
	(0.065)	(0.036)	(0.036)	(0.036)	(0.059)	(0.033)	(0.033)	(0.033)
Web	−0.175***	0.004	0.011	−0.009	0.063	−0.011	−0.013	−0.002
	(0.060)	(0.079)	(0.079)	(0.079)	(0.055)	(0.073)	(0.073)	(0.073)
Density	−0.005	0.001	0.000	0.001	−0.010	0.005	0.005	0.005
	(0.015)	(0.021)	(0.021)	(0.021)	(0.014)	(0.019)	(0.019)	(0.019)
Industry FE	Yes	Yes	Yes	Yes	Yes	Yes	Yes	Yes
Observations	868	868	868	868	868	868	868	868
R-squared	0.157	0.173	0.178	0.178	0.107	0.108	0.108	0.111

Robust standard errors are in parentheses. *** *p*<0.01, ** *p*<0.05, * *p*<0.1. The constant coefficient is not reported. Columns (1a) and (1b) report estimates from specification (1) for perceived stress and life satisfaction, respectively. The corresponding pair of estimates from specifications (2), and (3) are shown in columns (2a)–(2b) and (3a)–(3b), respectively. All regressions are performed using a linear regression model and are weighted with entropy balancing weights.

### Empirical design

We examine the impact of the COVID-19 pandemic on the well-being of entrepreneurs, as well as how this impact is moderated by subjective age and proximity to the local epicenter of the outbreak. We start by estimating the results for a model with only control variables (referred to as “base”). Specification (1) is measuring the general impact of COVID-19 on the well-being of new entrepreneurs:

(1) $$Well-being_{i}=\alpha_{1,1}+\alpha_{2,1}COVID-19_{i}+\alpha_{3,1}Stockholm+a_{4,1}Feel\;younger_{i}+X_{i}\Theta +u_{i}$$

where *Well-being* represents either perceived stress or life satisfaction, *COVID-19* is an indicator of whether the survey response was submitted after the outbreak of COVID-19 in week 12, *Stockholm* is an indicator of whether the respondent lives in Stockholm County, *Feel younger* is a binary variable indicating whether the respondent’s subjective age is lower than their chronological age, $X_{i}$ is a vector of the control variables that may impact well-being, and $u_{i}$ is the error term. In specification (1), the coefficient of interest is $\alpha_{2,1}$ , which captures the difference in well-being between individuals who responded to the survey before the COVID-19 outbreak and individuals who responded to the survey after the outbreak. The *Stockholm* and *Feel younger* coefficients ( $\alpha_{3,1}$ and $\alpha_{4,1}$ , respectively) capture the general relationship between well-being and living in Stockholm and between well-being and feeling younger, respectively.

To examine the moderating effect of subjective age (*illusion of age*) and proximity to the epicenter (*illusion of space*), we extend the specification 1 with an interaction term between *COVID-19* and *Feel younger* (specification 2) and between *COVID-19* and *Stockholm* (specification 3). The coefficient of each interaction term captures the additional effect of feeling younger (specification 2) or living in Stockholm (specification 3) following the onset of the pandemic, conditional on the general association between well-being and feeling younger or living in Stockholm.

(2) $$\begin{array}{l} Well-being_{i}=\alpha_{1,2}+\alpha_{2,2}COVID-19_{i}+\alpha_{3,2}Stockholm_{i}+a_{4,2}Feel\;younger_{i}\\ +\beta_{1}Stockholm_{i}\times COVID-19_{i}+X_{i}\Theta +u_{i} \end{array}$$

(3) $$\begin{array}{l} Well-being_{i}=\alpha_{1,3}+\alpha_{2,3}COVID-19_{i}+\alpha_{3,3}Stockholm_{i}+a_{4,3}Feel\;younger_{i}\\ +\beta_{2}Feel\;younger_{i}\times COVID-19_{i}+X_{i}\Theta +u_{i} \end{array}$$

All regressions are performed using linear regression models with robust standard errors and are weighted with entropy balancing weights (Hainmueller, 2012).

## Results and Discussion

Moving to the main regression analysis, Table 3 shows the results of specifications (1)–(3) for the sample of entrepreneurs. Columns (1a) and (1b) report estimates from specification (1) for perceived stress and life satisfaction, respectively. The corresponding pair of estimates from specifications (2) and (3) are shown in columns (2a)–(2b) and (3a)–(3b), respectively.

The COVID-19 estimate in Column (1a) shows that entrepreneurs who responded to the survey in the post-outbreak period report higher levels of perceived stress than those who responded to the survey before the outbreak. The magnitude of the coefficient represents a 0.26 unit increase in average perceived stress from the pre-COVID-19 specification 1. Dividing the coefficient by the mean of the dependent variable (2.308), we conclude that the estimated effect corresponds to an 11% increase in stress. The COVID-19 estimate in Column (1b) also shows a negative effect on life satisfaction, but the coefficient is not statistically significant. These findings provide support in favor of our baseline hypothesis that the COVID-19 outbreak is negatively correlated with entrepreneurs’ well-being in terms of stress. For the base estimation using stress as the outcome, the R-squared is 0.157 and when the main variables are included, the R-squared increases to 0.173 (when no interactions are made). With interactions the R-squared increases to 0.178. The corresponding R-squared values for the life satisfaction estimation are 0.107 and 0.108, respectively, showing a moderate increase. Yet, this is expected given the statistical insignificance of the variables throughout the estimations.

Unsurprisingly, those who feel younger are generally less stressed. In addition, the insignificant coefficient of *Stockholm* suggests that overall, there is no differences in well-being between entrepreneurs in Stockholm and entrepreneurs elsewhere in Sweden.

We now turn to the interaction terms in Columns (2a)–(3b). First, the negative estimate of the interaction term between *COVID-19* and *Feel younger* in column (2a) implies that the increase in stress level is smaller among entrepreneurs who feel younger than their chronological age compared to entrepreneurs whose subjective age is the same as or higher than their chronological age. This result indicates that feeling younger may moderate the overall negative effect of COVID-19 on entrepreneurs’ stress levels and is thus in line with Hypothesis 1. The positive coefficient of the interaction term between *COVID-19* and *Feel younger* in column (2b), where the outcome variable is life satisfaction, also signals a buffering impact of feeling younger on well-being, although the coefficient is statistically insignificant.

Second, the interaction terms between *COVID-19* and *Stockholm* in Columns (3a) and (3b), examine whether the effects of the pandemic on well-being are greater in the local epicenter of the outbreak, Stockholm, compared to the rest of the country. The positive coefficient of the interaction term in Column (3a) suggests that entrepreneurs in Stockholm experienced a larger increase in stress compared to entrepreneurs in other regions. This result is in line with Hypothesis 2, which stipulates a negative relationship between geographic distance to the local epicenter and the impact of the pandemic on well-being. The sign of the corresponding coefficient of the interaction term in Column (3b), where life satisfaction is the outcome, is in line with Hypothesis 2.

**Figure 1.**
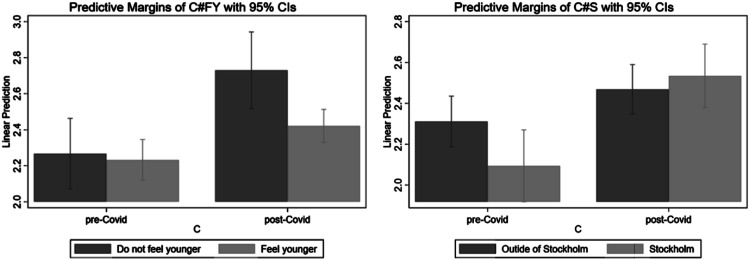
Predictive margins for stress of the interactions between feeling younger and COVID-19 (left) and Stockholm and COVID-19 (right). The sample consists of entrepreneurs in the survey.

**Figure 2.**
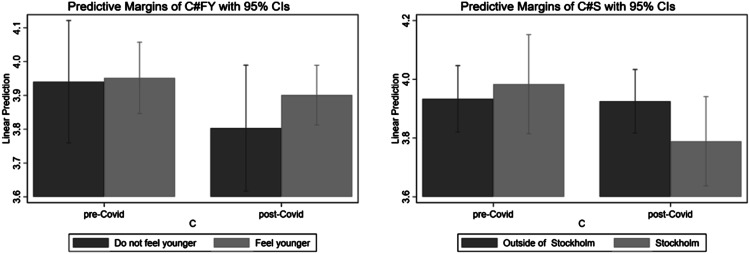
Predictive margins for life satisfaction of the interactions between feeling younger and COVID-19 (left) and Stockholm and COVID-19 (right). The sample consists of entrepreneurs in the survey.

To visualize these effects, Figures 1 and 2 plots the predicted values of stress and life satisfaction, respectively, for the interaction terms from specifications (2) and (3). Starting with Figure 1, the left chart focuses on the interaction between *COVID-19* and *Feel younger*. It shows that individuals had similar stress levels before the *COVID-19* outbreak, regardless of whether they feel younger (the bars have equal height). Although the stress level of both groups is higher after the pandemic outbreak (the height of both bars increases), the increase is relatively smaller for those who feel younger.

A similar story is depicted in the right chart, which plots the predicted values of stress for the different combinations of *COVID-19* and *Stockholm*. While entrepreneurs who live in the Stockholm region report lower stress levels before the outbreak relative to those who live outside Stockholm, the pattern reverses after the outbreak—those living in Stockholm report higher stress levels than those outside Stockholm.

The patterns are similar in Figure 2. The left chart shows no significant differences in life satisfaction between those who feel younger and those who do not feel younger before the COVID-19 outbreak. On the other hand, those who feel younger show higher rates of life satisfaction before the COVID-19 outbreak. The right chart shows that entrepreneurs in Stockholm had somewhat higher life satisfaction before the COVID-19 outbreak. The situation is reversed post COVID-19; Stockholm entrepreneurs report lower life satisfaction than those located elsewhere.

As for the control variables, the results show that older individuals generally experience lower stress levels and higher life satisfaction. Married individuals as well as those with higher income also report being more satisfied with their lives. Those with higher income also report lower stress levels in general. Entrepreneurs who had received sickness benefits during previous years report higher stress levels and lower life satisfaction. Moreover, females are more likely to report being stressed, though this is only significant at the 10% level. The coefficients of the rest of the control variables are statistically insignificant.

### Robustness Tests and Post-Hoc Analysis

We conduct four sets of analyses to strengthen the internal validity of the results by testing: (i) entrepreneurs versus employees; (ii) *illusion of age*: the stability of subjective age; (iii) *illusion of space*: the Stockholm effect versus density effect; and (iv) temporal selection in survey responses.

### Entrepreneurs Versus Employees

Our baseline hypothesis is that entrepreneurs are particularly subjected to stress during a major crisis. If this premise holds true, entrepreneurs should be more affected by the COVID-19 pandemic than employees. Thus, when we repeat the analysis for our survey group of employees, we expect to find smaller or insignificant effects on our outcomes of interest—stress and life satisfaction. The results are shown in Table 4. The *COVID-19* estimate in Column (1a), where the measure of well-being is stress level, has the expected sign (positive) but is small and insignificant. The corresponding estimate in Column (1b), where the measure of well-being is life satisfaction, is also insignificant. In addition, all interaction terms have insignificant estimates in Columns (2b)–(3b). These findings support the premise of our study; the COVID-19 outbreak has different impacts on entrepreneurs and employees, and the outbreak is likely to be particularly stressful for entrepreneurs compared to employees.

**Table 4.** Main Results for the Control Group of Employees.

	Stress	Life Satisfaction	Stress	Life Satisfaction	Stress	Life Satisfaction
	(1a)	(1b)	(2a)	(2b)	(3a)	(3b)
COVID-19	0.031	0.032	0.081	0.033	0.041	0.049
(C)	(0.093)	(0.084)	(0.158)	(0.143)	(0.102)	(0.093)
Feel younger	−0.209**	0.245***	−0.178	0.245**	−0.209**	0.244***
(FY)	(0.082)	(0.074)	(0.114)	(0.104)	(0.082)	(0.074)
Stockholm	−0.176*	−0.031	−0.176*	−0.031	−0.156	0.003
(S)	(0.098)	(0.089)	(0.098)	(0.089)	(0.130)	(0.118)
C*FY			−0.063	−0.001		
			(0.163)	(0.148)		
C*S					−0.037	−0.067
					(0.164)	(0.149)
Age	−0.042***	0.015***	−0.042***	0.015***	−0.042***	0.015***
	(0.004)	(0.004)	(0.004)	(0.004)	(0.004)	(0.004)
Female	0.150**	0.135*	0.148*	0.135*	0.150*	0.135*
	(0.076)	(0.069)	(0.076)	(0.069)	(0.076)	(0.069)
Education	−0.006	0.092***	−0.006	0.092***	−0.007	0.091***
	(0.032)	(0.029)	(0.032)	(0.029)	(0.032)	(0.029)
Married	0.033	0.275***	0.032	0.275***	0.032	0.274***
	(0.068)	(0.062)	(0.068)	(0.062)	(0.068)	(0.062)
Income	−0.063*	0.041	−0.063*	0.041	−0.063*	0.042
	(0.035)	(0.032)	(0.035)	(0.032)	(0.035)	(0.032)
Sick	0.114	−0.139**	0.113	−0.139**	0.113	−0.141**
	(0.075)	(0.068)	(0.075)	(0.068)	(0.075)	(0.068)
Web	−0.128	0.023	−0.128	0.023	−0.126	0.027
	(0.099)	(0.090)	(0.099)	(0.090)	(0.100)	(0.091)
Density	0.036	0.000	0.036	0.000	0.036	0.000
	(0.022)	(0.020)	(0.022)	(0.020)	(0.022)	(0.020)
Industry FE	Yes	Yes	Yes	Yes	Yes	Yes
Observations	696	696	696	696	696	696
R-squared	0.175	0.111	0.176	0.111	0.175	0.111

Robust standard errors are in parentheses. *** *p*<0.01, ** *p*<0.05, * *p*<0.1. The constant coefficient is not reported. Columns (1a) and (1b) report estimates from specification (1) for perceived stress and life satisfaction, respectively. The corresponding pair of estimates from specifications (2), and (3) are shown in columns (2a)–(2b) and (3a)–(3b), respectively. All regressions are performed using a linear regression model and are weighted with entropy balancing weights.

### Stability of Subjective Age: Out-of-Sample Validation Tests

An important assumption that underlies our analysis of the role of subjective age is that “feeling younger” is a stable attribute that is unaffected in the short term by external events or emotional changes such as increased stress levels. If, for example, increased levels of stress caused by the COVID-19 crisis induce people to feel older, we may misinterpret the role of subjective age as a moderator. To explore the stability of subjective age, we conducted an independent online survey on a representative sample of Swedish citizens aged 40–80.^8^ The survey had a longitudinal design with three waves sent out at 1-week intervals. In each of the three waves, participants were asked to answer the same question about subjective age as the participants in our main survey were asked: “Regardless of your actual age, how old do you feel?” We then analyze within-individual changes in subjective age using a percentage gap definition of subjective age and binary indicators for feeling younger than one’s chronological age, feeling older or the same age. A total of 100 individuals answered all three survey waves.

We find that that the subjective age distribution resembles the corresponding distribution in the main survey. On average across all survey waves, 76% feel younger than their actual age, 11% feel older and 13% feel the same. The corresponding shares in the main survey are 75%, 20%, and 4%, respectively. The mean percentage gap in subjective age is slightly larger in the new survey (14%) compared to the main survey (10%), but the variation is similar (standard deviation of 15% in both). The similarities between the two independent surveys arguably strengthen the internal and external validity of our results that pertain to subjective age.

**Figure A3 -:**
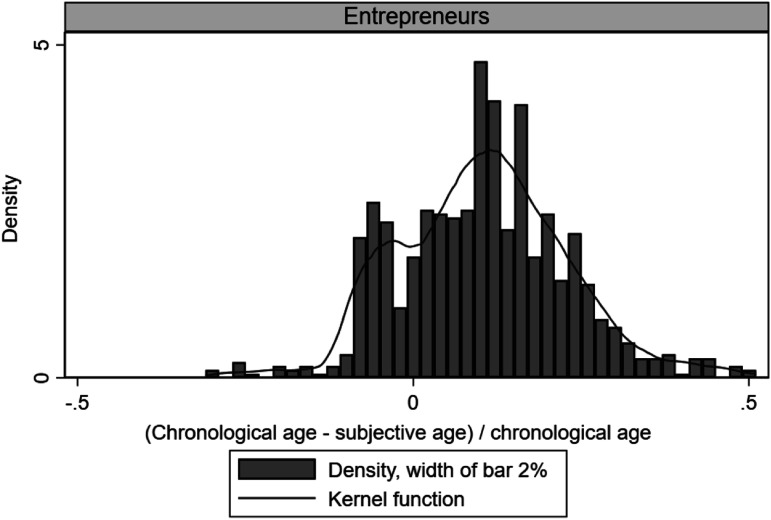
Distribution of the percentage gap in subjective age in the main survey. Outliers for the top and bottom 1% of the respondents are excluded.

**Figure A4 -:**
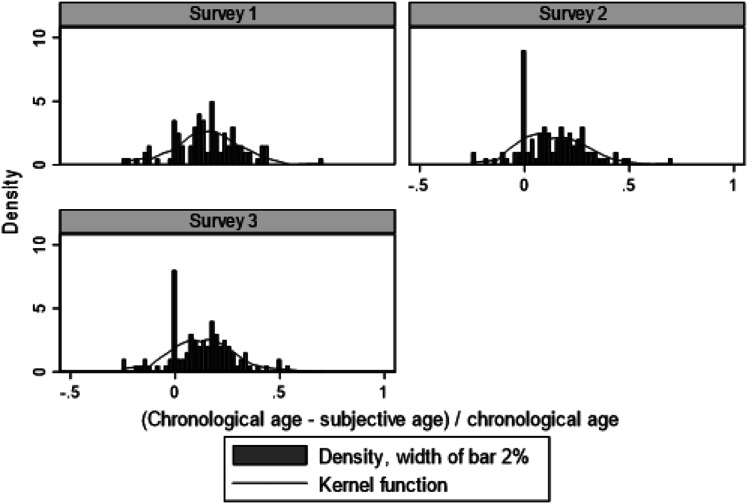
Distribution of the percentage gap in subjective age in the out-of-sample survey.

Turning to the variation in subjective age over time, notably, as many as 85% of the respondents remain in the same subjective age category across all three waves. We further plot the full distribution of the percentage gap from each survey wave (Figure A4). The most visually significant movement between survey waves 1 and 2 is from feel younger than one’s actual age to feel the same age. Specifically, the feel younger share drops from 83% to 71% while the share of feel the same increases from 7% to 18%. The corresponding movements between the second and third survey waves are much smaller. Overall, however, the three distributions appear very similar and are similar to the corresponding distribution in the main sample (Figure A3).

**Figure A5 –:**
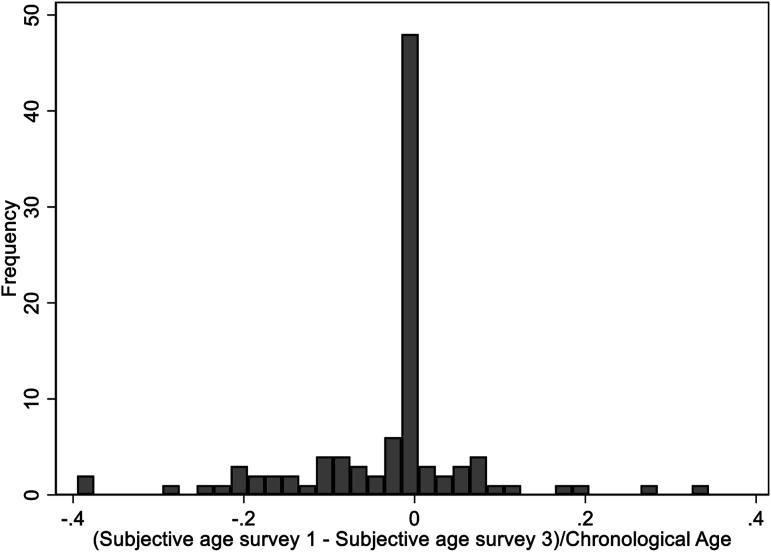
Difference in subjective age between the first and third survey wave in the out-of-sample survey, normalized by chronological age.

To further explore the variation in subjective age, we plotted the difference in subjective age between the first and third surveys, divided by chronological age (Figure A5). The spike at 0 indicates that 1) more than 30% of the respondents report exactly the same subjective age in the three waves, and 2) individuals are on average more likely to transition to a younger subjective age.

In sum, our out-of-sample survey demonstrates that most respondents do not change subjective age category (feel younger, feel the same and feel older) and that within-individual changes in subjective age are slightly skewed toward younger ages, yet to a minor extent.^9^ We therefore consider a binary classification of feeling younger as a stable attribute. In addition, a binary classification reduces the influence of unreasonably large differences between subjective and chronological age (outliers) and statistical noise from random variation in subjective age.

Nevertheless, to further validate the main results in Table 3 we replace the binary variable with a proportional discrepancy measure of subjective age (Table A2). Since this variable is sensitive to the inclusion of outliers, we have dropped the 1^st^ and 100^th^ percentiles (15 observations).^10^ Similar issues with outliers have been reported by Shane et al. (2019) who truncate them, and by Westerhof and Barrett (2005) who also drop the 1^st^ and 100^th^ percentile. The results remain consistent and confirm our hypotheses.

### Stability of Subjective Age: Further Robustness Tests

We also use our original survey to address the concern that subjective age may be a non-stable personal attribute. First, we quantify the relationship between the *Feel younger* variable and several key variables. These key variables include a dummy variable for the onset of the pandemic *(COVID-19),* a dummy variable indicating whether the individual lives in *Stockholm*, and a continuous variable for chronological age. The insignificant estimates of *COVID-19* in most of the columns of the top row of Table A3 strengthen our conclusion that subjective age identity does not seem to be unduly affected by the pandemic, at least not in the short term. The coefficient of *Stockholm* is also insignificant, indicating that people in Stockholm on average do not feel younger than people elsewhere in the country when controlling for chronological age. The fact that the likelihood of feeling younger increases with age is in line with previous research documenting a negative relationship between chronological and subjective age (Ye & Post, 2020; Rubin & Berntsen, 2006; Mirucka et al., 2016).

The interaction term between *COVID-19* and Age (C*Age) addresses the concern that the relationship between chronological age and subjective age may differ between early and late survey respondents. A change in this relationship could indicate the presence of a third, unobserved factor which in turn may be correlated with well-being. The third interaction term between *Age* and *Stockholm* examines whether the relationship between chronological age and subjective age is different between entrepreneurs in Stockholm and entrepreneurs in the rest of the country. A significant estimate would make it problematic to generalize the feeling younger results to other localities, that is, limit the external validity of the results. Reassuringly, all three estimates are small and statistically insignificant.

Another concern related to our subjective age measure is the possibility that the results are driven by a general age effect. To explore this, we re-estimate specifications (1)–(3) after interacting *COVID-19* and *Stockholm* with chronological age instead of *Feel younger* (thus far, chronological age has only been included as a control variable). *Feel younger* is still included in the regressions as a control variable.^11^ The estimates show that our main results are not driven by chronological age; instead, feeling younger appears to be the factor that mitigates the effects of the crisis on stress.

Finally, we have explored whether the effect of subjective age and feeling younger changes with chronological age. A younger age identity may matter more for older individuals’ well-being during stressful events. To explore this, we interacted binary indicators for various age cut-offs with *Feel younger* and *COVID-19*. The results indicate that the effect of *Feel younger* did not vary across the age distribution after the COVID-19 outbreak.

### Illusion of Space

While Stockholm was the local epicenter of the pandemic in Sweden in the early spring of 2020, the economic impacts felt in Stockholm are not any direr than those felt in other regions of Sweden (see the section *The COVID-19 Outbreak in Sweden*). In practice, there may be other factors beyond proximity to the local epicenter related to living in Stockholm driving changes in well-being during our survey period. One such factor could be population density—individuals living in other metropolitan areas farther away from the epicenter may have been similarly affected as individuals living in Stockholm. First, recall that we include a rich set of controls in our regressions, such as population density and a dummy for living in Stockholm, both of which had insignificant coefficients in most cases. To further strengthen our argument, we perform an additional sensitivity analysis with “placebo” counties.

Specifically, we re-estimate the main results in Table 3 treating the second largest Swedish county, *Västra Götaland*, where the city of Gothenburg is located, as the geographic entity of interest. Individuals living in Stockholm are hence dropped from the sample. The estimates are shown in Table A4. If the coefficients on the COVID-19 interaction terms are significant, the results would indicate that the effect on well-being is due to population density—a “big city” effect—rather than proximity to the epicenter. Västra Götaland is an interesting comparison since the region has the highest share of short-term work allowances per capita in Sweden (Ekonomifakta, 2020). Thus, while the medical risks may have been higher in Stockholm, the economic consequences and implications for entrepreneurs were likely worse in Gothenburg. Reassuringly, the coefficients of the interaction terms are insignificant.

### Temporal Selection in Survey Responses

Our empirical strategy implies that we compare the well-being of entrepreneurs who responded to the survey before the COVID-19 outbreak to the well-being of those who responded after the outbreak. Consequently, we interpret any decline in well-being as a direct result of the worsening COVID-19 situation. Obviously, there may be factors that are correlated with both well-being and the propensity to respond quickly or slowly to a survey. For example, if individuals of lower socioeconomic status tend to respond later, and if these individuals generally experience more stress than their peers of higher socioeconomic status, we may falsely attribute an increase in stress levels during the COVID-19 period to the crisis when in actuality, the observed increase in stress levels reflects natural changes in the composition of respondents. We control for selection based on such *observables* by including a rich set of controls in our regressions (see the previous section).^12^

By conditioning on observables, our empirical approach relies on the assumption that there are no *unobservable* factors driving differences in well-being that are dependent on the timing of the survey responses. The potential selection based on unobservables is arguably more difficult to address. We verify the validity of the identifying assumption by running placebo estimations using only “pre-COVID-19” observations with an imaginary “post-COVID-19” cut-off. Specifically, we re-estimate specifications (1)–(3) for the sample of entrepreneurs using responses from week 8 (Feb. 17–23) and weeks 9–11 (Feb. 24–Mar. 15) as pre- and “post-COVID-19” observations, respectively. If the speed of responding to the survey is positively related to well-being, meaning that our post-COVID-19 group would have worse measures of well-being regardless of the outbreak, we should also observe this pattern among those who responded to the survey before the outbreak. Reassuringly, the results in Table A5 show that the coefficients for the placebo *COVID-19* variable are insignificant. Additionally, all COVID-19 placebo interactions have insignificant coefficients.

## Discussion and Conclusion

In this paper, we sought to understand the effects of a major crisis—such as the COVID-19 pandemic—on the well-being of entrepreneurs. We further strive to understand how entrepreneurs can maintain their well-being during such a crisis. We identify two forms of psychological detachment that could mitigate the negative effects of the crisis on entrepreneurs’ well-being: *illusion of age* (feeling younger than one’s chronological age) and *illusion of space* (being geographically distant from the crisis). Our main findings are threefold and suggest that the positive illusions we identify are effective moderators, with a few caveats. First, we find that the outbreak of COVID-19 in Sweden has had a negative effect on the well-being of entrepreneurs, as proxied by an increase in reported stress levels. Second, we find that feeling younger mitigates the negative effects of the COVID-19 outbreak on stress levels. Third, our results show that living outside the local epicenter of the outbreak, that is, in Stockholm County, mitigates the negative effects of COVID-19 on stress levels.

In a supplementary analysis, we have also sought to further test our underlying assumption, namely that the effects of the COVID-19 outbreak differ between entrepreneurs and employees, and that the outbreak is likely to be a larger stressor for entrepreneurs compared to employees. In line with this idea, we find no significant effect of the COVID-19 outbreak on a sample of employees of similar age.

### Implications for Entrepreneurship Research on Well-Being

Our work provides several contributions to entrepreneurship research on well-being. By identifying the COVID-19 outbreak as a critical work-related stressor for entrepreneurs, our research contributes to the emerging research on entrepreneurs’ work stressors (Wach et al., 2020; Kollmann et al., 2019). While past research on work stressors has mainly considered employees’ experience in their work environment—such as work-overload or work complexity—or focused on generic stressors frequent to being an entrepreneur (Kollmann et al., 2019), we show that a major crisis, such as the outbreak of the COVID-19 pandemic, can be a stressor for entrepreneurs. The results of our supplementary analysis on the sample of employees (which do not show a significant effect of the COVID-19 outbreak on employees’ well-being) provide additional evidence of our claim. In addition, because the emerging research on the well-being of entrepreneurs has been largely conducted before the recent COVID-19 crisis (Lerman et al., 2020), it is likely to depict a brighter picture of entrepreneurship than what may be the reality. Thus, our study helps counterbalance this picture by providing evidence of the increased stress that entrepreneurs perceive after the COVID-19 outbreak, which is one of the potential dark sides of entrepreneurship (Spivack & McKelvie, 2018).

However, even if COVID-19 negatively impacts entrepreneurs’ stress levels, it does not seem to have a discernible effect on their life satisfaction, at least not in the short term. There are several potential explanations for this finding, the perhaps most straightforward relates to the stream of research considering hedonic entrepreneurial well-being as comprising different components, including general evaluations of one’s life (i.e., life satisfaction) as well as negative affect through stress (Diener et al., 1999). It is possible that at the outbreak of a crisis, these components of well-being are separable aspects.

Our study proposes that psychological detachment from the crisis can potentially assist entrepreneurs in alleviate stress during turbulent times. Specifically, our research considers two forms of detachments—*illusion of age* and *illusion of space*. These are positive illusions, which are not necessarily conscious or accurate assessments, but that may still help entrepreneurs psychologically detach from the COVID-19 outbreak and the corresponding work-related stressors. While we cannot fully disentangle these specific effects, our tests suggest that entrepreneurs who feel younger and entrepreneurs who are located further from the epicenter of the crisis were less stressed following the crisis. This is a contribution because we uncover boundary conditions to the impact of a major and sudden crisis on entrepreneurial well-being that have not been examined to date. In addition, by theorizing about psychological detachment from the crisis as a mechanism that can help entrepreneurs to maintain well-being, we shed new light on how some entrepreneurs can maintain positive functioning despite adversity. In doing so we add to the research on how entrepreneurs build resilience and react after major crises (Farny et al., 2019; Shepherd and Williams, 2014, 2020).

### Implications for Research Beyond the Entrepreneurship Literature

Our study also adds to research beyond the entrepreneurship literature. Research on subjective age and well-being has shown that feeling younger can play an important role in alleviating the negative effects of stressful events such as cancer (Boehmer, 2007) and Post-Traumatic Stress Disorder (Palgi, 2016; Shrira et al., 2018). Although we study a rather specific setting, our findings align with these theories and concur with the notion that subjective age might also help individuals dissociate from a traumatic event, such as the sudden outbreak of COVID-19. In addition, an understanding of the role of subjective age—as opposed to chronological and/or biological age—with regards to entrepreneurial well-being is particularly critical in modern society, which tends to celebrate youth and in which older individuals are confronted with age stereotypes, age norms, and feeling socially devalued (Weiss & Lang, 2012; Levy, 2009).

Our study is also related to economic geography, in which the role of physical proximity and geographical distance between economic actors and to different resources and inputs has been extensively studied (Tobler, 1970; Lavoratori et al., 2020). We add to this literature by scrutinizing how entrepreneurs who are geographically distant from the epicenter of a crisis detach themselves from the crisis. As conceptualized in our paper, proximity works through the way economic actors perceive their reality; these perceptions could subsequently influence their behavior and outcomes. These mechanisms are often neglected in studies analyzing the role of geographical distance.

### Practical Implications

Our study has practical implications, particularly for the current debate on policy tools and interventions during a crisis. Our finding that the crisis had an almost direct effect on the well-being of entrepreneurs but not on employees, in terms of perceived stress, calls for government policies that are directed toward entrepreneurs and are implemented swiftly. The finding that entrepreneurs are more affected by this kind of crisis compared to employees favors policies that account for firm heterogeneity, for example, policies could target a certain group of firms, such as new or small firms. As most new jobs in OECD countries are created in small and medium-sized firms,^13^ the well-being of the entrepreneurs who establish and run these firms is crucial to overall society.

Our research is also relevant for entrepreneurship educators. There are several educational programs that aim to foster and enhance entrepreneurship. These programs and initiatives should prepare entrepreneurs for the challenges of the entrepreneurial process, including the increased stress both during a major crisis as well as in its aftermath. This education could be done in multiple ways. One method could be to provide realistic accounts and narratives of these phenomena, moving away from narratives of the heroic figure of the entrepreneur (Cacciotti et al., 2016). In addition, educators could focus on the critical role of detachment from work-related stressors. In their career, entrepreneurs will need to take steps to facilitate detachment to mitigate stress levels and other factors that negatively impact their overall well-being.

### Limitations and Direction for Future Research

As with all studies, our work is not without limitations, and some of the issues we discuss can be used as direction and input for future studies. Our research design builds on cross-sectional data from which we draw inference from between-individual variation rather than within-individual variation. We conducted a battery of post-hoc analyses and robustness tests to strengthen the internal validity of the results and to rule out alternative explanations. However, even though the findings of our study are rigorous, the cross-sectional nature of the data can lead to questions of endogeneity. One potential question may result from reverse causality between perceived stress and feeling younger: the observed negative relationship between feeling younger and stress may not reflect a buffering impact of feeling younger if occasional periods of stress make some people feel older. A related question is simultaneity bias, implying that perceived stress and feeling younger might be jointly determined. Such bias may arise if the outbreak of COVID-19 coincided with other life-changing events that simultaneously influenced perceived stress and feeling younger. Examples of such events could be divorce or partners losing their jobs, which we were unable to control for. Although we include several control variables and run a series of post-hoc and robustness tests, we are aware that the general concern of endogeneity is not fully addressed given the cross-sectional nature of data. Therefore, we cannot infer causality but correlations in our study and encourage future studies to develop research designs for causal inference (Anderson et al., 2019).

Another limitation relates to the moderate increase in *R*^2^ when including the interaction variables in the estimations, which could indicate limited practical effect size for the moderating role of the illusion of age and the illusion of space on the relationship of COVID-19 and well-being. Future studies should broaden our understanding of other moderators for the well-being of entrepreneurs during sudden and major crises.

The survey and the register data used in our empirical design originates from, like many other studies, one country; as such the findings may not be generalizable to other countries. Even though the Swedish government diverged from other countries in how they responded to the COVID-19 outbreak, businesses in Sweden experienced similar declines in aggregate consumer spending as countries that enforced lockdowns (Sheridan et al., 2020). In addition, Juranek et al. (2020) show that the labor markets of all Nordic countries were severely hit by the pandemic and that Sweden performed only slightly better than its neighbors. They find similar results for mobility using Google’s COVID-19 Community Mobility Reports, i.e., that mobility decreased in all countries in the first weeks after the outbreak of the initial phase, yet slightly less in Sweden. As the economic shock mainly seems to be a result of the virus outbreak rather than the implemented restrictions (Sheridan et al., 2020), our findings are applicable to other business owners experiencing the same contraction in economic activity. More research is albeit still warranted in other settings to further investigate the external validity of our findings.

COVID-19 is first and foremost a medical crisis, and the economic recession is the consequence of necessary public health measures (Baldwin & di Mauro, 2020). As such, the pandemic allows us to study a crisis coupled with high levels of uncertainty in terms of its duration and scope. Future research can investigate the key role of governments in providing financial assistance to, in many cases, otherwise well-functioning firms, and how such assistance measures themselves may impact the well-being of entrepreneurs.

Our study is restricted to individuals between the ages of 40 and 80, leading to a limited sample that does not capture the lower age distribution of entrepreneurs. However, given the age distribution of novice entrepreneurs in Sweden, in which the peak occurs around the age of 40 (Backman & Karlsson, 2018; Holmquist & Sundin, 2017), we captured the majority of new entrepreneurs. In combination with our research question, the cut-off age of 40 makes empirical and theoretical sense (Westerhof & Barrett, 2005; Ye & Post, 2020; Barrett & Montepare, 2015). However, future research can analyze younger cohorts and how they behave during a crisis.

In this paper, we control for the ownership type of the firm and separate limited liability firms and sole proprietorship since most novice entrepreneurs employ one of these two ownership structures. There is room for more in-depth analysis on how ownership type relates to the well-being of entrepreneurs and possible policy recommendations.

Our paper explores the moderating role of the two forms of detachment from the crisis—feeling younger and/or being located far from the epicenter of the crisis. Although we provide validation of our hypotheses, we cannot conclusively prove the mechanisms at play. We hope to encourage further research on these mechanisms.

There are other fruitful ways in which our study can be expanded. The highlighted temporal difference across well-being in terms of life satisfaction and stress levels needs further attention from both a theoretical and empirical perspective. In addition, as emphasized in other studies (Stephan, 2018), more research is needed into the eudemonic aspects of well-being rather than only focusing on the hedonic aspects of well-being. Building on the different psychological distances (Trope & Liberman, 2010; Bar-Anan et al., 2007), research could also be conducted on detachment strategies that entrepreneurs can employ to detach themselves from a crisis. Lastly, our study shows that employees were less affected by the crisis in terms of well-being compared to entrepreneurs. However, we need more knowledge on how employees are affected; for example, employees in new firms could plausibly react differently to a crisis than employees in more mature firms. Future research might extend our work on comparing entrepreneurs and employees during the COVID-19 pandemic. For instance, we focus on well-being, but other studies could compare the two groups across other aspects such as differences in resilience, positive emotions, collaboration with other internal and external stakeholders, job satisfaction, utility derived from their occupational choice, pandemic fatigue, emotional exhaustion, and potential effects on income and income growth.

## Figures and Tables

**Figure A2:**
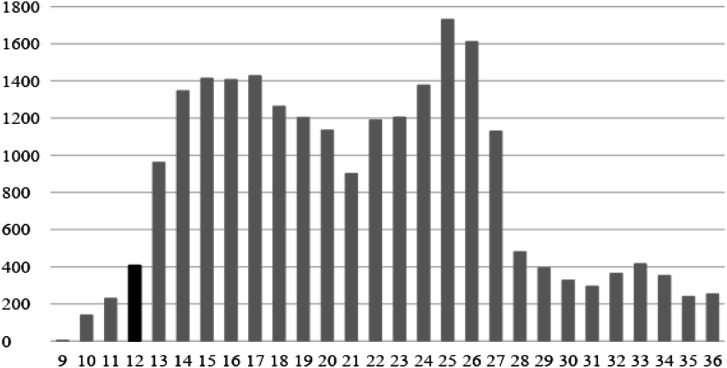
Number of COVID-19 cases in Stockholm county, on a weekly basis, week 12 (March 16–22) is highlighted in the figure as this is the week used in the empirical design. Source: https://experience.arcgis.com/experience/19fc7e3f61ec4e86af178fe2275029c5, accessed on October 14, 2020.

## Notes

1. In a global context, the most pertinent example of the COVID-19 epicenter is Wuhan in China. A local epicenter is defined as a region in a country that has experienced the worst outcome in terms of the number of cases and the number of deaths is documented in many countries, for example, Milan in Italy, Madrid in Spain, Santiago in Chile, and Stockholm in Sweden.

2. https://www.government.se/articles/2020/04/strategy-in-response-to-the-covid-19-pandemic/, accessed on October 14, 2020.

3. March 12: the Swedish government restricted gatherings to no more than 500 people. March 16: the government issued an edict that individuals over the age of 70 should stay at home and encouraged individuals in Stockholm county to work from home if possible. March 17: universities and high schools switched to teaching online. March 19: travel restrictions were implemented. March 29: the government restricted gatherings to no more than 50 people. April 1: visits to elderly care homes were banned. https://www.di.se/nyheter/detta-har-hant-sverige-under-coronakrisen-dag-for-dag/, accessed on October 14, 2020.

4. https://experience.arcgis.com/experience/09f821667ce64bf7be6f9f87457ed9aa/page/page_0/, accessed on October 14, 2020.

5. https://experience.arcgis.com/experience/19fc7e3f61ec4e86af178fe2275029c5, accessed on October 14, 2020. See Appendix Figure A2 for weekly statistics of the number of cases in the Stockholm county.

6. The use of subjective age in empirical studies has increased over the past decade. Some studies have examined participants over several years; they find that the measurement is stable over longer time horizons (Cleveland et al., 1997; Uotinen et al., 2006; Kleinspehn-Ammerlahn et al., 2008), albeit with some individual variations. Other studies have instead focused on day-to-day changes in subjective age (Kotter-Grühn et al., 2015; Goecke & Kunze, 2020). Taken together, these studies suggest that although micro-fluctuations occur, over time these fluctuations are smoothed. The observed fluctuations are also of such a scale that they do not drastically alter the qualitative aspect of subjective age. Someone who feels younger than her chronological age will still feel younger even if she is stressed or having a bad day health wise.

7. On average, the survey participants feel approximately 10% younger than they are (see Table 1 and Figure A3 for the complete distribution of the percentage gap).

8. The survey was an online survey, conducted by Origo Group during 26 March – 12 April 2021.

9. Bellingtier and Neupert (2019) find that subjective age is more subject to changes among older people. Because of our focus on the age group 40–80, subjective age is, if anything, likely higher in other contexts. However, we find no significant differences along the age dimension in the propensity to change subjective age category, nor in the magnitude of subjective age changes (results available upon request).

10. The reported subjective age (in relation to their chronological age) for the dropped observations displayed a highly skewed age perception. For example, the respondents that were removed felt up to 89% younger or 81% older than their chronological age.

11. The results from this analysis can be requested from the authors.

12. We examine how individual characteristics vary across the timing of the survey responses. We find that entrepreneurs who respond late, on average, are younger, are more likely to be female, are less likely to be retired, have higher incomes, and are less likely to run an incorporated business. Education and sectoral experience are, however, unrelated to the timing of the survey responses.

13 http://www.oecd.org/cfe/leed/1918307.pdf, accessed December 4, 2020.

## Appendix

**Table A1:** Sample Selection, Respondents Versus Targeted Population. The Table Shows the Means of the Covariates.

	Entrepreneurs^a^			Employees^b^		
Variable	Sample	Population	Difference	Sample	Population	Difference
Age	55.589	53	2.593**	53.845	53.274	−0.571
Education	4.223	3.956	2.267**	3.85	3.658	0.192**
Female	0.354	0.385	−0.031*	0.559	0.508	0.051**
Married	0.657	0.591	0.066**	0.562	0.546	0.016
Income	4583.726	4185.71	398.016*	3815	31,718.382	96.574
Sick	0.272	0.324	−0.052**	0.302	0.325	−0.023
Stockholm	0.131	0.138	−0.007	0.084	0.089	−0.005
Obs.	1332	4498		715	2,717,504	

** *p*<0.01, * *p*<0.05.

a The population of entrepreneurs consists of all entrepreneurs to whom we sent the survey. A t-test done on the subset and the entire population to whom we sent the survey.

b The population consists of all employees in the same age group in 2016. The sample is weighted with design weights to account for the stratification on age when drawing a sample from the population for the survey. The t-test in this case is done between the subsample and the full population.

**Table A2:** Results for Entrepreneurs With Interactions When Subjective Age Is Calculated as Proportional Rather Than a Binary Measure and the 1% Outliers Are Dropped.

	Stress	Life Satisfaction	Stress	Life Satisfaction	Stress	Life Satisfaction
	(1a)	(1b)	(2a)	(2b)	(3a)	(3b)
COVID-19	0.238***	−0.046	0.328***	−0.072	0.135	0.007
(C)	(0.075)	(0.069)	(0.088)	(0.081)	(0.086)	(0.079)
Subjective age	−0.948***	0.461**	−0.512	0.334	−0.960***	0.467**
(SA)	(0.220)	(0.201)	(0.314)	(0.288)	(0.219)	(0.201)
Stockholm	−0.043	−0.078	−0.037	−0.080	−0.214*	0.009
(S)	(0.085)	(0.078)	(0.085)	(0.078)	(0.110)	(0.100)
C*SA			−0.858*	0.249		
			(0.442)	(0.405)		
C*S					0.306**	−0.155
					(0.124)	(0.114)
Age	−0.034***	0.019***	−0.035***	0.019***	−0.035***	0.019***
	(0.004)	(0.003)	(0.004)	(0.003)	(0.004)	(0.003)
Female	0.106	0.091	0.107	0.090	0.098	0.095
	(0.066)	(0.060)	(0.066)	(0.060)	(0.066)	(0.060)
Education	0.002	0.006	0.004	0.005	0.002	0.006
	(0.030)	(0.028)	(0.030)	(0.028)	(0.030)	(0.028)
Incorporated	0.041	0.057	0.038	0.058	0.036	0.060
	(0.063)	(0.058)	(0.063)	(0.058)	(0.063)	(0.058)
Married	−0.107*	0.229***	−0.106*	0.229***	−0.112*	0.232***
	(0.060)	(0.054)	(0.059)	(0.054)	(0.059)	(0.054)
Income	−0.041**	0.058***	−0.043**	0.059***	−0.040**	0.058***
	(0.019)	(0.017)	(0.019)	(0.017)	(0.019)	(0.017)
Sick	0.135**	−0.179***	0.123*	−0.175***	0.139**	−0.181***
	(0.066)	(0.061)	(0.067)	(0.061)	(0.066)	(0.061)
Industry Exp.	0.010	0.011	0.011	0.011	0.006	0.013
	(0.036)	(0.033)	(0.036)	(0.033)	(0.036)	(0.033)
Web	0.007	0.028	0.016	0.026	−0.005	0.035
	(0.080)	(0.073)	(0.080)	(0.073)	(0.080)	(0.073)
Density	−0.003	0.016	−0.004	0.016	−0.003	0.016
	(0.021)	(0.019)	(0.021)	(0.019)	(0.021)	(0.019)
Industry FE	Yes	Yes	Yes	Yes	Yes	Yes
Observations	853	853	853	853	853	853
R-squared	0.179	0.116	0.183	0.116	0.185	0.118

Robust standard errors are in parentheses. *** *p*<0.01, ** *p*<0.05, * *p*<0.1. The constant coefficient is not reported. Columns (1a) and (1b) report estimates from specification (1) for perceived stress and life satisfaction, respectively. The corresponding pair of estimates from specifications (2) and (3) are shown in columns (2a)–(2b) and (3a)–(3b), respectively. All regressions are performed using a linear regression model and are weighted with entropy balancing weights.

**Table A3:** Estimations When “Feel Younger” Is the Dependent Variable to Show That Feeling Younger Is a Stable Attribute That Does Not Change Over Time and That Is Not Affected by Events.

Dependent Variable: Feel Younger	(1)	(2)	(3)	(4)	(5)
COVID-19 (C)	0.019	0.020	0.019	0.064	0.360
	(0.032)	(0.031)	(0.031)	(0.045)	(0.219)
Age		0.010***	0.011***	0.011***	0.014***
		(0.002)	(0.002)	(0.002)	(0.003)
Stockholm (S)			0.018	0.082	0.084*
			(0.033)	(0.060)	(0.047)
C*S				−0.002	
				(0.068)	
C*Age					−0.006
					(0.004)
Female				−0.004	−0.001
				(0.036)	(0.036)
Education				−0.012	−0.013
				(0.016)	(0.016)
Incorporated				0.012	0.014
				(0.033)	(0.033)
Married				−0.049	−0.045
				(0.032)	(0.032)
Income				0.008	0.009
				(0.010)	(0.010)
Sick				−0.031	−0.028
				(0.035)	(0.036)
Industry Exp.				−0.029	−0.028
				(0.019)	(0.019)
Web				0.073*	0.068*
				(0.040)	(0.041)
Density				−0.024**	−0.024**
				(0.011)	(0.011)
Industry FE	No	No	No	Yes	Yes
Observations	868	868	868	868	868
R-squared	0.000	0.034	0.035	0.065	0.067

Robust standard errors are in parentheses*** *p*<0.01, ** *p*<0.05, * *p*<0.1. All regressions are performed using a linear regression model and are weighted with entropy balancing weights.

**Table A4:** Location Interactions Using Västra Götaland Instead of Stockholm

Variables	Stress	Life Satisfaction	Stress	Life Satisfaction	Stress	Life Satisfaction
	(1a)	(1b)	(2a)	(2b)	(3a)	(3b)
COVID-19	0.123	0.009	0.144	0.043	0.145	−0.004
(C)	(0.098)	(0.088)	(0.160)	(0.144)	(0.104)	(0.093)
Feel younger	−0.176**	0.073	−0.162	0.096	−0.177**	0.074
(FY)	(0.084)	(0.075)	(0.120)	(0.108)	(0.084)	(0.075)
V. Götaland	0.183*	−0.063	0.183*	−0.063	0.247*	−0.103
(VG)	(0.098)	(0.088)	(0.098)	(0.088)	(0.142)	(0.128)
C*FY			−0.027	−0.044		
			(0.164)	(0.148)		
C*VG					−0.115	0.072
					(0.185)	(0.166)
Age	−0.038***	0.016***	−0.038***	0.016***	−0.038***	0.016***
	(0.005)	(0.004)	(0.005)	(0.004)	(0.005)	(0.004)
Female	0.164*	0.110	0.163*	0.109	0.166*	0.109
	(0.085)	(0.076)	(0.085)	(0.076)	(0.085)	(0.076)
Education	0.006	0.007	0.006	0.007	0.005	0.007
	(0.036)	(0.033)	(0.036)	(0.033)	(0.036)	(0.033)
Incorporated	0.058	0.042	0.058	0.042	0.057	0.043
	(0.077)	(0.069)	(0.077)	(0.070)	(0.077)	(0.070)
Married	−0.089	0.227***	−0.089	0.227***	−0.090	0.228***
	(0.074)	(0.066)	(0.074)	(0.066)	(0.074)	(0.066)
Income	−0.046**	0.075***	−0.046**	0.075***	−0.045*	0.074***
	(0.023)	(0.021)	(0.023)	(0.021)	(0.023)	(0.021)
Industry Exp.	−0.007	0.020	−0.007	0.020	−0.008	0.021
	(0.045)	(0.041)	(0.045)	(0.041)	(0.045)	(0.041)
Sick	0.080	−0.139*	0.080	−0.139*	0.081	−0.139*
	(0.082)	(0.073)	(0.082)	(0.073)	(0.082)	(0.073)
Web	−0.069	0.039	−0.068	0.042	−0.068	0.038
	(0.102)	(0.092)	(0.102)	(0.092)	(0.102)	(0.092)
Density	−0.021	−0.007	−0.021	−0.007	−0.021	−0.007
	(0.027)	(0.024)	(0.027)	(0.024)	(0.027)	(0.024)
Industry FE	Yes	Yes	Yes	Yes	Yes	Yes
Observations	597	597	597	597	597	597
R-squared	0.183	0.104	0.183	0.104	0.184	0.104

Robust standard errors are in parentheses. *** *p*<0.01, ** *p*<0.05, * *p*<0.1. The constant coefficient is not reported. Columns (1a) and (1b) report estimates from specification (1) for perceived stress and life satisfaction, respectively. The corresponding pair of estimates from specifications (2) and (3) are shown in columns (2a)–(2b) and (3a)–(3b), respectively. All regressions are performed using a linear regression model and are weighted with entropy balancing weights.

**Table A5:** Using a Placebo Cut-off for the COVID-19 Outbreak Among the Sample of Entrepreneurs

Variables	Stress	Life Satisfaction	Stress	Life Satisfaction	Stress	Life Satisfaction
	(1a)	(1b)	(2a)	(2b)	(3a)	(3b)
COVID-19	0.083	−0.123	0.030	0.041	0.160	−0.122
(C)	(0.085)	(0.078)	(0.160)	(0.147)	(0.100)	(0.092)
Feel younger	−0.056	−0.016	−0.092	0.097	−0.055	−0.016
(FY)	(0.098)	(0.090)	(0.135)	(0.124)	(0.098)	(0.090)
Stockholm	−0.259**	0.060	−0.257**	0.053	−0.112	0.061
(S)	(0.125)	(0.115)	(0.125)	(0.115)	(0.159)	(0.147)
C*FY			0.073	−0.226		
			(0.187)	(0.172)		
C*S					−0.268	−0.003
					(0.182)	(0.168)
Age	−0.033***	0.024***	−0.033***	0.024***	−0.034***	0.024***
	(0.006)	(0.005)	(0.006)	(0.005)	(0.006)	(0.005)
Female	0.136	0.044	0.132	0.057	0.136	0.044
	(0.097)	(0.089)	(0.098)	(0.090)	(0.097)	(0.089)
Education	−0.067	0.001	−0.066	−0.001	−0.062	0.001
	(0.046)	(0.042)	(0.046)	(0.042)	(0.046)	(0.042)
Incorporated	0.131	−0.033	0.134	−0.039	0.126	−0.033
	(0.091)	(0.084)	(0.091)	(0.084)	(0.091)	(0.084)
Married	−0.072	0.088	−0.074	0.094	−0.070	0.088
	(0.087)	(0.080)	(0.088)	(0.080)	(0.087)	(0.080)
Income	−0.047	0.108***	−0.047	0.109***	−0.046	0.108***
	(0.030)	(0.027)	(0.030)	(0.027)	(0.030)	(0.027)
Sick	0.166*	−0.131	0.166*	−0.130	0.167*	−0.131
	(0.098)	(0.090)	(0.098)	(0.090)	(0.098)	(0.090)
Industry Exp.	−0.029	0.032	−0.027	0.027	−0.027	0.032
	(0.054)	(0.049)	(0.054)	(0.049)	(0.053)	(0.049)
Web	−0.172	0.424	−0.164	0.397	−0.140	0.424
	(0.374)	(0.344)	(0.375)	(0.344)	(0.374)	(0.345)
Density	0.021	0.000	0.020	0.002	0.018	0.000
	(0.030)	(0.028)	(0.030)	(0.028)	(0.030)	(0.028)
Industry FE	Yes	Yes	Yes	Yes	Yes	Yes
Observations	390	390	390	390	390	390
R-squared	0.184	0.146	0.184	0.150	0.189	0.146

Robust standard errors are in parentheses. *** *p*<0.01, ** *p*<0.05, * *p*<0.1. The constant coefficient is not reported. Columns (1a) and (1b) report estimates from specification (1) for perceived stress and life satisfaction, respectively. The corresponding pair of estimates from specifications (2) and (3) are shown in columns (2a)–(2b) and (3a)–(3b), respectively. All regressions are performed using a linear regression model and are weighted with entropy balancing weights.
